# MRI texture feature repeatability and image acquisition factor robustness, a phantom study and *in silico* study

**DOI:** 10.1186/s41747-020-00199-6

**Published:** 2021-01-19

**Authors:** Joshua Shur, Matthew Blackledge, James D’Arcy, David J. Collins, Maria Bali, Martin O’Leach, Dow-Mu Koh

**Affiliations:** 1 Department of Radiology, The Royal Marsden NHS Foundation Trust, Downs Road, Sutton, London, Surrey SM2 5PT UK; 2 CRUK Cancer Imaging Centre, The Institute of Cancer Research and The Royal Marsden NHS Foundation Trust, London, UK

**Keywords:** Magnetic resonance imaging, Phantoms (imaging), Reproducibility of results, Radiomics, Texture analysis

## Abstract

**Purpose:**

To evaluate robustness and repeatability of magnetic resonance imaging (MRI) texture features in water and tissue phantom test-retest study.

**Materials and methods:**

Separate water and tissue phantoms were imaged twice with the same protocol in a test-retest experiment using a 1.5-T scanner. Protocols were acquired to favour signal-to-noise ratio and resolution. Forty-six features including first order statistics and second-order texture features were extracted, and repeatability was assessed by calculating the concordance correlation coefficient. Separately, base image noise and resolution were manipulated in an *in silico* experiment, and robustness of features was calculated by assessing percentage coefficient of variation and linear correlation of features with noise and resolution. These simulation data were compared with the acquired data. Features were classified by their degree (high, intermediate, or low) of robustness and repeatability.

**Results:**

Eighty percent of the MRI features were repeatable (concordance correlation coefficient > 0.9) in the phantom test-retest experiment. The majority (approximately 90%) demonstrated a strong or intermediate correlation with image acquisition parameter, and 19/46 (41%) and 13/46 (28%) of features were highly robust to noise and resolution, respectively (coefficient of variation < 5%). Agreement between the acquired and simulation data varied, with the range of agreement within feature classes between 11 and 92%.

**Conclusion:**

Most MRI features were repeatable in a phantom test-retest study. This phantom data may serve as a lower limit of feature MRI repeatability. Robustness of features varies with acquisition parameter, and appropriate features can be selected for clinical validation studies.

**Supplementary Information:**

The online version contains supplementary material available at 10.1186/s41747-020-00199-6.

## Key points

- Magnetic resonance imaging (MRI) texture analysis is being increasingly utilised.
- Most MRI features are repeatable in a phantom test-retest experiment.
- Most MRI features are sensitive to image noise and resolution.

## Background

Radiomics refers to the extraction of quantitative imaging features from anatomical and functional imaging data [1, 2]. Within radiomics, texture analysis is typically combined with data mining and machine learning with the goal of delivering precision medicine. In oncology, radiomics has shown the potential to describe tumour pathology and predict tumour behaviour such as response to therapy and overall survival [3]. These analyses are driven by the hypothesis that variations in texture correlate with tumour phenotype or its biological expression [3]. For example, radiomics has been investigated in most tumour types, such as breast cancer [4], lung cancer [5], and gliomas where it has shown utility in tumour grading and survival prediction [6].

It is recognised that challenges to radiomics include standardisation of image acquisition, feature extraction, and segmentation [1]. The use of robust quantitative data is particularly necessary in larger multi-centre studies which might include variations in operator, location, measurement systems, and techniques. To better understand the robustness and generalizability of any radiomic discoveries, analyses should therefore include an objective assessment of the reproducibility and repeatability of radiomics features.

Initially applied to photomicrographs and satellite data [7], and within medical imaging to computed tomography (CT) and positron emission tomography (PET) data, texture analysis has been more recently applied to magnetic resonance imaging (MRI), which has brought its own unique challenges and opportunities. Unlike CT, in which tissue contrast is determined by atomic number, physical density, and photon energy, MRI tissue contrast arises from the interactions of tissue properties, such as proton density and longitudinal and transverse relaxation times, with image acquisition parameters, such as the echo and repetition times [8]. The cellular microenvironment influences the MRI signal by the way it modifies the motion of water molecules [8]. The increased possibilities for contrast in MRI lead to the potential for increased variability in the derived radiomics features, together with the possibility that correlations discovered may reflect differences in acquisition protocol rather than clinically useful findings. Thus, with MRI, the need for standardisation is crucial [9].

Typically, a radiomic signature is validated by applying it prospectively to a larger independent dataset, better when across multiple sites. Estimates of the repeatability and stability of radiomic features are essential both to aid interpretation of cohort findings and to enable application of texture features to monitoring of changes in individual patients. Little data exists regarding repeatability of MRI features in a test-retest scenario, and there is a paucity of data addressing sensitivity of those features to acquisition parameter [10].

The aim of this study was two-fold. Firstly, we aimed to quantify MRI feature robustness with acquisition parameters that influence image noise and resolution using simulations and experimental data. Secondly, we aimed to derive a lower limit on MRI radiomic feature repeatability by performing a test-retest experiment. The relationship between signal-to-noise ratio (SNR), image resolution, and radiomic feature repeatability was assessed.

## Methods

### MRI phantom and acquisition protocol

A test-retest study was performed on a commercial water MRI phantom (Siemens Healthcare 5,300 mL nickel sulphate serial number 2147, Erlangen, Germany) and a tissue phantom (leg of lamb, New Zealand) using a clinical 1.5-T Siemens Magnetom Aera scanner (Siemens Healthcare, Erlangen, Germany). Research ethics board approval was not required.

The water phantom and tissue phantom were positioned in the magnet socentre at room temperature. The long axis of the cylindrical phantom and the long bone of the tissue phantom were aligned with the *z*-axis of the magnet. Oil-filled fiducial markers were included to aid repositioning.

Sequence parameters typical of clinical T1- and T2-weighted sequences were used to acquire images, and the number of excitations (NEX) and image matrix size varied to acquire images with reduced SNR and resolution (Table 1).

**Table 1:** Parameters for water and tissue phantom image acquisition in the test-retest study

	A	B	C	D	E	F
Weighting	T2	T2	T2	T1	T1	T1
Repetition time (ms)	3,000	3,000	3,000	323	323	323
Echo time (ms)	82	82	82	4.76	4.76	4.76
Number of excitations	32	1	1	32	1	1
Matrix	256 × 256	256 × 256	128 × 128	256 × 256	256 × 256	128 × 128
Slice thickness (mm)	5	5	5	5	5	5
Pixel spacing (mm)	1.18	1.18	2.34	1.17	1.17	2.34
Echo train length	24	24	24	2	2	2
Bandwidth (kHz)	300	300	300	455	455	455
Flip angle (°)	150	150	150	70	70	70
Field of view (mm)	243 × 300	243 × 300	243 × 300	225 × 300	225 × 300	225 × 300
Slice spacing (mm)	5.5	5.5	5.5	5.5	5.5	5.5

Sequences A and D provided high signal-to-noise ratio and high spatial resolution, while sequences B and E provided reduced signal-to-noise ratio, and C and F also reduce the resolution

Following the low-SNR and low-resolution scans, the water and the tissue phantoms were removed from the MRI scanner and then immediately repositioned in the magnet socentre. The time interval between test and retest was less than 5 min.

For each arm of the test-retest study, five non-contiguous axial slices were obtained.

### Texture feature extraction

Radiomic feature calculation was performed using a custom-built script in Matlab (2017a, The MathWorks Inc., Natick, MA, USA). Second-order texture features were calculated from the grey-level co-occurrence matrix (GLCM) and grey-level run-length matrix (GLRLM) matrices, as described by Haralick et al. [7] and Galloway [11], using the method described in the paper by Aerts et al. [3].

Fourteen first-order statistics and 32 second-order texture features were calculated giving 46 radiomic features in total, and these are outlined in Table 2. The GLCM and GLRLM matrices describe respectively the frequency of grey-level combinations occurring in immediately adjacent pixels, and the frequency of the lengths of consecutive runs of pixels having the same grey level, respectively. The features were calculated in each direction separately and the two-dimensional GLCM and two-dimensional GLRLM were then averaged over all directions and normalised using the method described in Aerts et al. [3].

**Table 2:** List of computed statistic (S) and texture (T) features

Number	Feature	Abbreviation
1.	Energy	S.En
2.	Entropy	S.Ent
3.	Kurtosis	S.Kur
4.	Maximum	S.Max
5.	Mean	S.Mean
6.	Mean absolute deviation	S.MAD
7.	Median	S.Med
8.	Minimum	S.Min
9.	RMS	S.RMS
10.	Range	S.Ran
11.	Skewness	S.Sk
12.	Standard deviation	S.Std
13.	Uniformity	S.Un
14.	Variance	S.Var
15.	Autocorrelation	T.Aut
16.	Cluster prominence	T.Clp
17.	Cluster shade	T.Cls
18.	Cluster tendency	T.Clt
19.	Contrast	T.Con
20.	Correlation	T.Cor
21.	Difference entropy	T.Den
22.	Dissimilarity	T.Dis
23.	Energy	T.En
24.	Entropy	T.Ent
25.	Grey-level non uniformity	T.GLNU
26.	High grey-level run emphasis	T.HGLRU
27.	Homogeneity 1	T.Hom1
28.	Homogeneity 2	T.Hom2
29.	Informational measure correlation 1	T.IMC1
30.	Informational measure correlation 2	T.IMC2
31.	Inverse difference moment normalised	T.IDMN
32.	Inverse difference normalised	T.IDN
33.	Inverse variance	T.IV
34.	Long run emphasis	T.LRE
35.	Long run high grey-level emphasis	T.LRHGLE
36.	Long run low grey-level emphasis	T.LRLGLE
37.	Low grey-level run emphasis	T.LoGLRU
38.	Maximum probability	T.MP
39.	Run length non uniformity	T.RLNU
40.	Run percentage	T.RP
41.	Short run emphasis	T.SRE
42.	Short run high grey-level emphasis	T.SRHGLE
43.	Short run low grey-level emphasis	T.SRLGLE
44.	Sum average	T.SA
45.	Sum entropy	T.SE
46.	Sum variance	T.SV

Features were calculated from fixed regions of interest (ROI) for the tissue and water phantoms. The ROI coordinates and size were identical for each acquisition and for each arm of the test-retest. Any variations in object sampling between the test and retest are therefore due to deviations in phantom positioning, which sets a lower bound on repeatability since accurate repositioning of patients is more challenging than for phantoms.

**Fig. 1:**
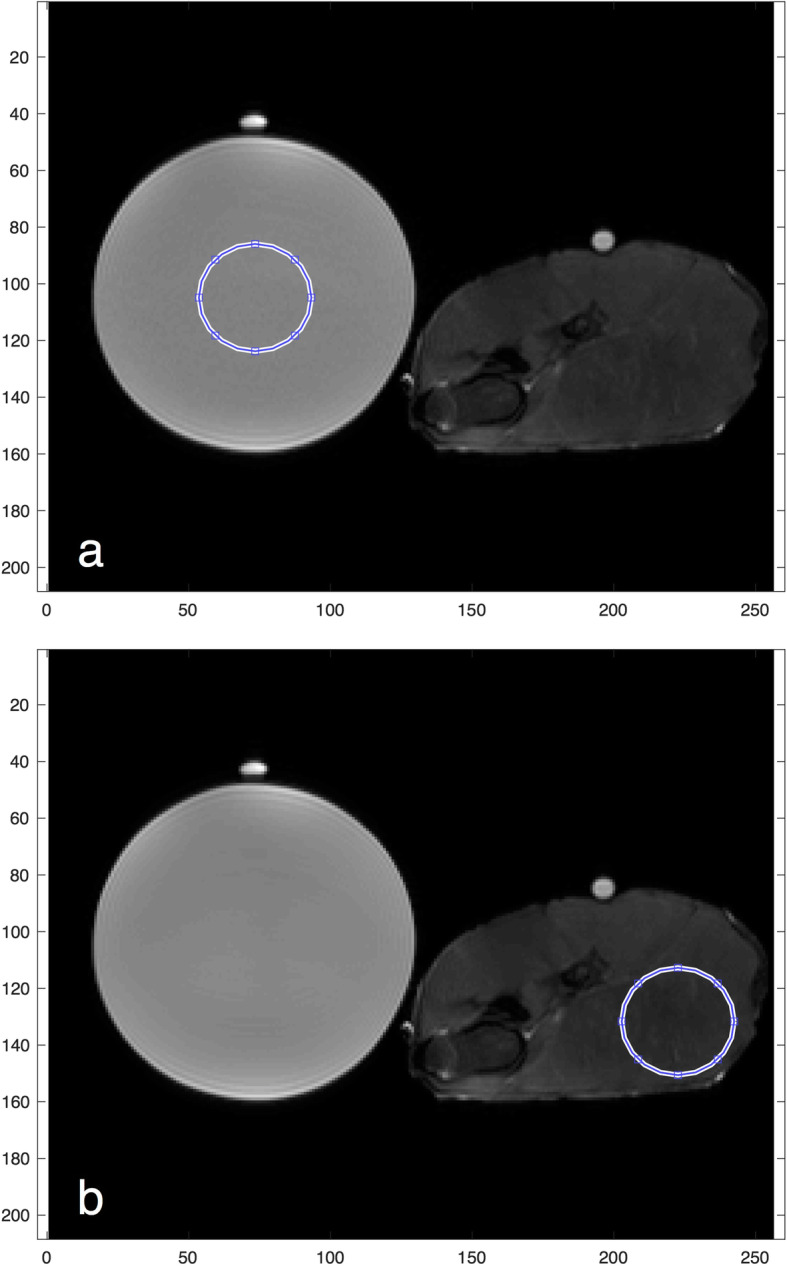
**a**, **b** Example of regions of interest (ROIs) used to calculate texture features for the water phantom (**a**) and tissue phantom (**b**). Identical circular ROIs were placed in the centre of the water phantom and in a homogenous muscle group of the tissue phantom

Circular ROIs were chosen within the centre of the water phantom and within a uniform muscle group of the tissue phantom to ensure the image texture was similar across the ROI (Fig. 1a, b). The ROI size was adjusted with matrix size so that the object region included in the ROI was kept constant.

Prior to calculating the GLCM, the data from each ROI was normalised by subtracting the mean and dividing by the standard deviation (SD) of pixel values in each ROI. This ensures that any differences in feature values are not due to variations in pixel value mean or SD. The data were then quantised into 16 discrete, uniformly spaced grey-level bins before GLCM calculation. The number of bins was chosen as it is within the 99.9% confidence interval for the *z* scores.

### Simulations of image noise and matrix size

Simulations were used to assess feature robustness across a wider range of noise and resolution than was feasible on the phantom measurements. The image data acquired with NEX equal to 32 were used as the base image from which to simulate images with different noise levels and resolution by adding synthetic noise and interpolating the voxel size.

The image noise and SNR were measured using the image difference method of Dietrich et al. [12] by using two independent images acquired using protocols B (for T2-weighted imaging) and E (for T1-weighted imaging) described in Table 1. The SNR values were 3.75 and 4.69 for the T2-weighted and T1-weighted sequences, respectively, which are sufficiently above 3 implying that the Rician noise essentially has a Gaussian distribution [13].

Images simulating the effect of different numbers of excitations were therefore generated by adding Gaussian noise to the base images acquired using protocols A and D described in Table 1, which we considered as being noise-free. The added noise standard deviations were the noise values estimated using the difference method, scaled by 1/square root (NEX) for NEX = 1, 7, 13, 19, 25, or 31.

Using the high SNR data for T2-weighted images, the image resolution was reduced with bicubic interpolation and antialiasing using the “imresize” function in Matlab, which is a recognised method of adjusting image resolution [14, 15]. Using a base image resolution of 256 pixels, output resolution was 256/*r* where *r* = 1 to 6, for 6 discrete output resolution levels in total. Features were then extracted for each of the 6 simulated noise and resolution levels.

A quantitative measure of feature robustness with respect to noise and resolution was calculated from the T2 data, the percentage coefficient of variation (%COV), as described by the QIBA (Quantitative Imaging Biomarkers Alliance), and its Terminology Working Group [16]. This is a measure of the spread of feature values, normalised to the mean value when noise or resolution is varied:

1 $$ \% COV= SD/ mean $$

where *SD* and mean are the standard deviation and mean value of the texture features over repeated measurements. The *%COV* describes how much a given feature will vary as noise or resolution is perturbed, under the ranges defined in these experiments and is an estimate of the magnitude of variation. It is commonly reported at the 95% precision limit, or conversely with a cutoff of 5% [16].

Assuming a linear relationship, a separate measure of the strength of dependence of feature values on noise and resolution was obtained using Pearson’s correlation coefficient between individual features and noise (via NEX) and resolution (matrix size).

### Test-retest

A measure of feature robustness with test-retest was defined by the concordance correlation coefficient (CCC) [17], which is commonly used to assess agreement in a test-retest scenario within medical imaging and therefore was chosen as a suitable metric:

2 $$ CCC=\frac{2\rho {\sigma}_x{\sigma}_y}{{\sigma_x}^2+{\sigma_y}^2+{\left({\mu}_x-{\mu}_y\right)}^2} $$

where *μ*, *ρ*, and *σ* are the means, correlation coefficient, and standard deviations of the two variables, respectively. Agreement was defined as poor, moderate, substantial, and near-perfect for a CCC of < 0.90, 0.9 ≤ CCC < 0.95, 0.95 ≤ CCC < 0.99, and > 0.99, respectively [18].

## Results

Table 3 summarises robustness of features with noise and resolution. Table 4 summarises linear correlation of the 46 individual features with noise and resolution. Table 5 summarises feature repeatability in the test-retest experiment.

**Table 3:** Robustness of texture features as a function of noise and resolution

Robustness	High	Mid	Low
Noise	1, 2, 4, 5, 7, 9, 15, 20, 24–26, 31, 32, 37, 40–42, 44, 45	3, 6, 8, 10, 12, 13, 16, 18, 21–23, 27–30, 33–36, 38, 39, 43, 46	11, 14, 17, 19
Resolution	5, 7, 9, 15, 21, 26, 30–32, 40, 41, 42, 44,	2–4, 6, 8, 12, 14, 18, 20, 22, 24, 27, 28, 33–37, 43, 45, 46	1, 10, 11, 13, 16, 17, 19, 23, 25, 29, 38, 39

For the number identifying features, see Table 2. High robustness was defined with %COV < 5, mid robustness with 5 < %COV < 30, and low robustness with %COV > 30

*%COV* Percentage coefficient of variation

**Table 4:** Texture features linear correlation with noise and resolution

Correlation	High (*r* > 0.8)	Mid (0.8 > *r* > 0.2)	Low (*r* < 0.2)
Noise	21, 22, 28–30, 32, 33, 35, 36	2, 3, 6, 8, 10, 11–20, 23–27, 31, 34, 37-46	1, 4, 5, 7, 9
Resolution	2, 13, 15, 16, 19, 22–24, 27–35, 38–41, 44, 45	1, 3, 4, 6, 8, 10–12, 14, 18, 21, 25, 26, 36, 42, 43, 46	5, 7, 9, 17, 20, 37

Low correlation implies texture features have no linear dependence and were therefore invariant to noise or resolution

*r* Correlation coefficient

**Table 5:** Texture features repeatability in the test-retest experiment

Repeatability	Poor (CCC < 0.9)	Moderate (0.9 ≤ CCC < 0.95)	Substantial (0.95 ≤ CCC < 0.99)	Near perfect (CCC > 0.99)
Test-retest for T1-weighted imaging	8, 16, 17, 20, 25, 34, 35, 36	18, 23, 33, 38, 43, 46	14, 19, 21, 22, 24, 29, 40, 41, 45	1, 2, 3, 4, 5, 6, 7, 9, 10, 11, 12, 13, 15, 26, 27, 28, 29, 30, 31, 32, 37, 39, 42, 44
Test-retest for T2-weighted imaging	3, 11, 15, 26, 35, 36, 37, 42, 43	10, 16, 18, 38, 44, 46	4, 14, 17, 19, 23, 29	1, 2, 5, 6, 7, 8, 9, 12, 13, 20, 21, 22, 24, 25, 27, 28, 30, 31, 32, 33, 34, 39, 40, 41, 45

*CCC* Concoradance correlation coefficient

**Fig. 2:**
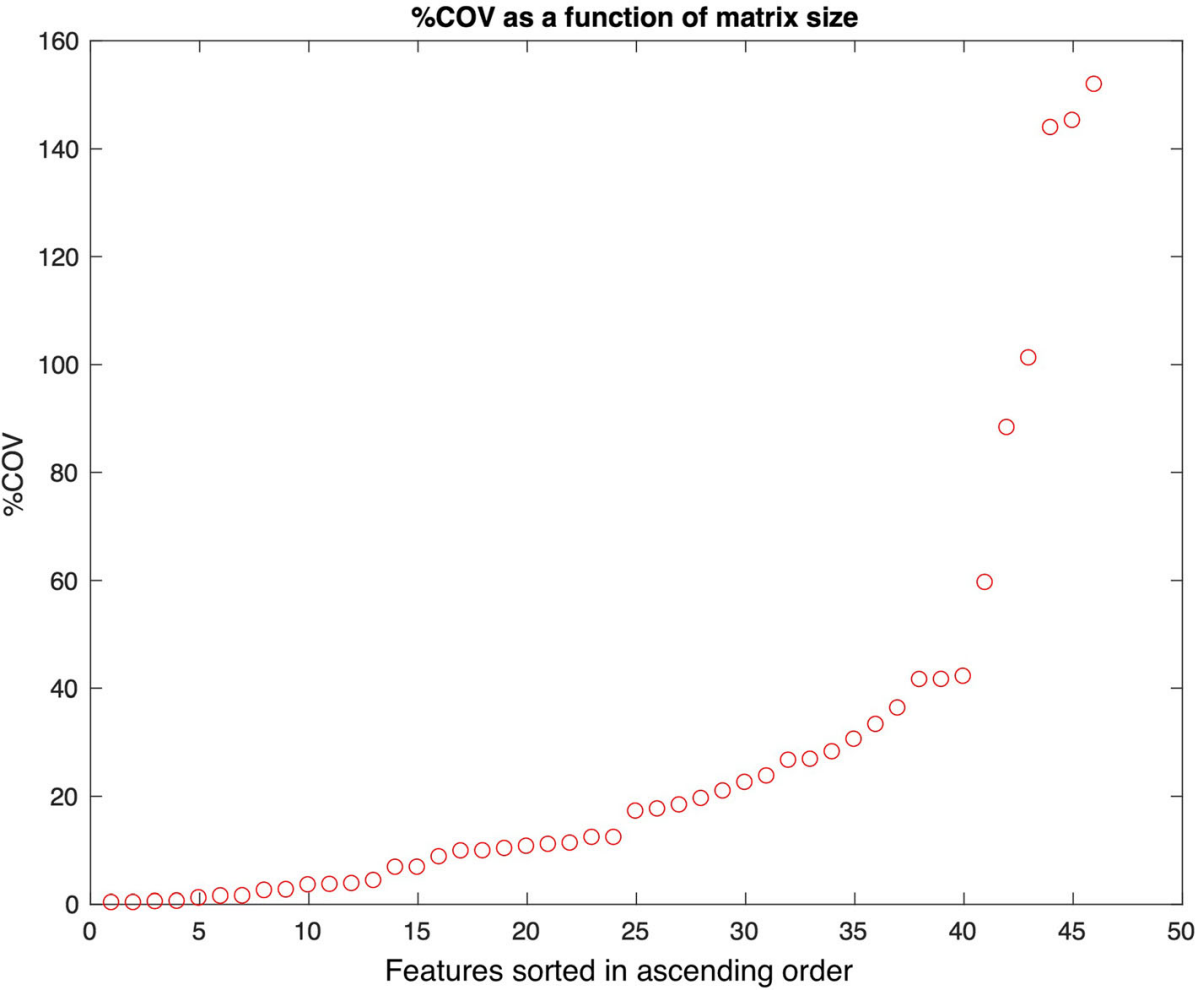
Percentage coefficient of variation (%COV) as a function of spatial resolution (matrix size) for 46 features, (14 statistics and 32 texture features) sorted in ascending order. Of 46 features, 12 demonstrated a low robustness (%COV > 30)

**Fig. 3:**
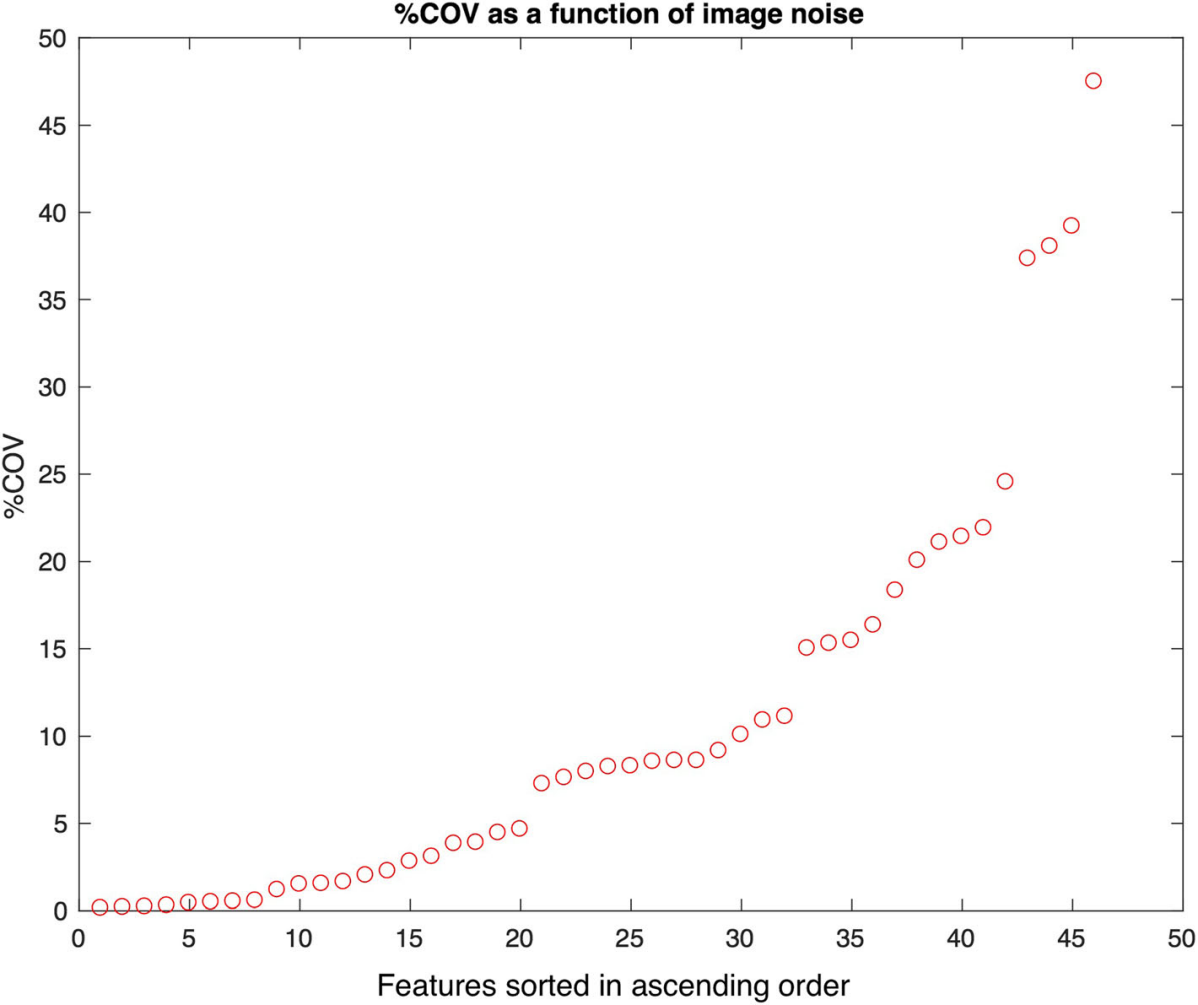
Percentage coefficient of variation (%COV) as a function of noise for 46 features (14 statistics and 32 texture features), sorted in ascending order. Of 46 features, 4 demonstrated a low robustness (%COV > 30)

Percentage COV as a function of image noise and resolution for the 46 features are shown in Figs. 2 and 3, sorted in ascending order. With a %COV cutoff at 5%, 19/46 (41%) and 13/46 (28%) of features were found to have high robustness with noise and resolution, respectively. With a %COV < 5 cutoff, 11/46 (24%) of features were highly robust to both noise and resolution (S.Mean, S.Med, S.RMS, T.Aut, T.HGLRU, T.IDMN, T.IDN, T.RP, T.SRE, T.SRHGLE, T.SA).

**Fig. 4:**
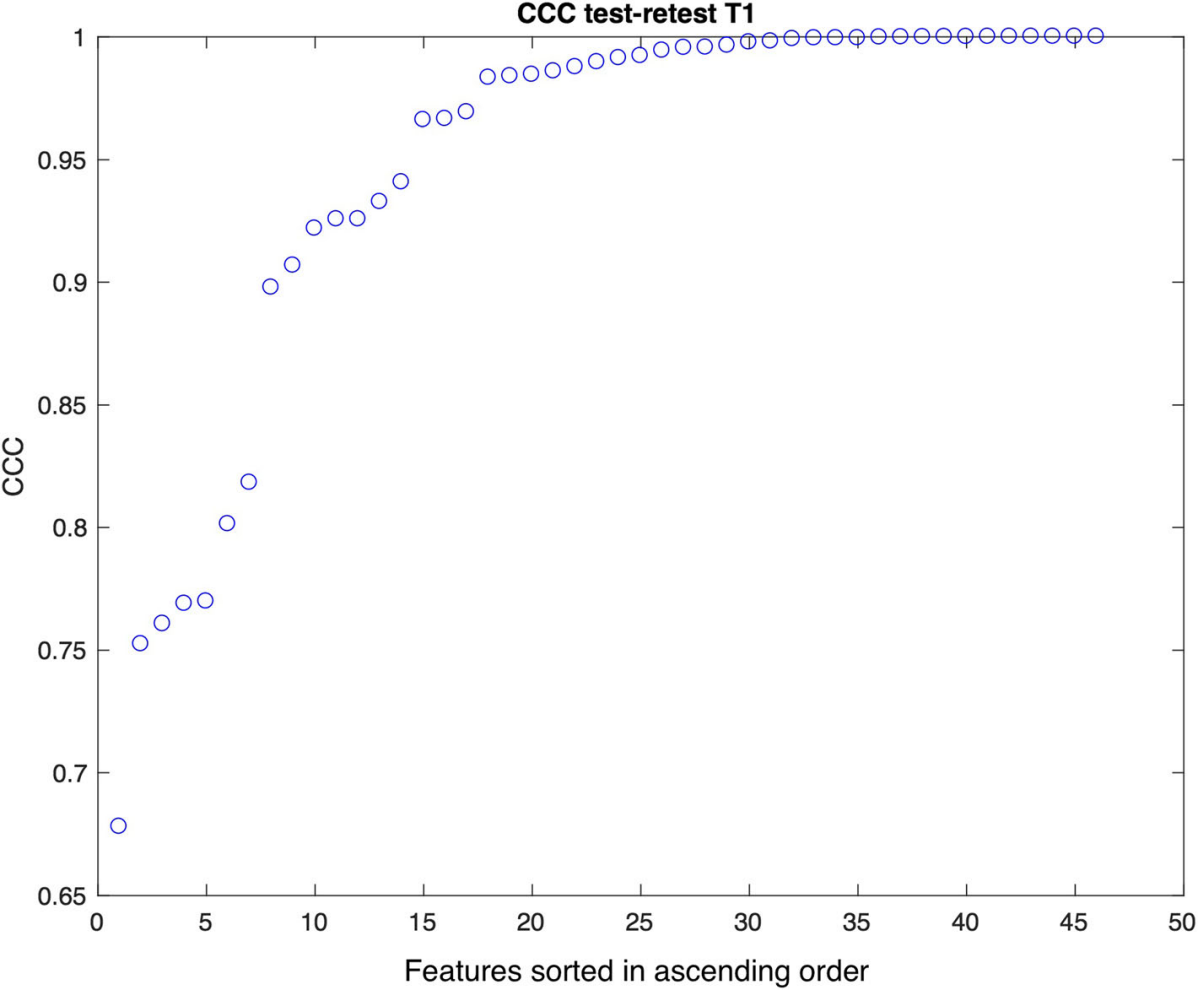
T1-weighted imaging. Concordance correlation coefficient (CCC) for 46 features (14 statistics and 32 texture features) in the test-retest experiment, sorted in ascending order

**Fig. 5:**
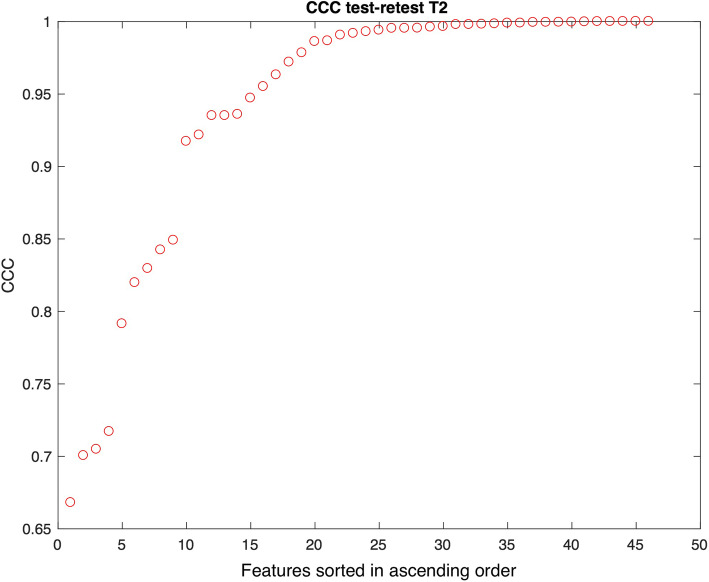
T2-weighted imaging. Concordance correlation coefficient (CCC) for 46 features (14 statistics and 32 texture features) in the test-retest experiment, sorted in ascending order

The results in terms of CCC of the test-retest experiment are demonstrated in Fig. 4 for T1-weighted imaging and Fig. 5 for T2-weighted imaging, sorted in ascending order. A CCC of 1 represents perfect agreement while a 0 value implies no agreement between test and retest results. Using a cutoff of CCC > 0.9, the majority of features demonstrate either moderate, substantial, or almost perfect repeatability: 38/46 (83%) for T1-weighted imaging and 36/46 (78%) for T2-weighted imaging, respectively. Three texture features demonstrated low repeatability for both T1- and T2-weighted imaging (T.Cls, TLRHGLE, and T.RLGLE).

Feature noise correlation data are found in Supplementary Figure S1 for both the tissue phantom (red dot) and water phantom (blue cross) for 5 adjacent image slices for 46 features. Supplementary Figure S2 demonstrates the same data but for features as a function of resolution, varying over *n* = 2 to 7 (1/2 to 1/7th base resolution).

The acquired resolution data and simulation data were in agreement in many cases, for example, the commonly used texture feature energy (texture features 23) and entropy (texture features 24); however, others demonstrated less agreement or even non-agreement (for example, texture features 19, contrast). Better agreement was observed for the resolution data than for the noise data. Agreement between the acquired and simulation data in general tended not to be dependent on feature class. This was demonstrated by a spread of features between the different classes: 1st order statistics (1–14), GCLM-derived features (15–33), and GLRLM features (34–46) demonstrating agreement between the acquired and simulation data. The lowest agreement was found in 11% of GLCM features for the water phantom noise experiment, and the highest agreement was found in 92% of GLRLM features for the tissue phantom resolution experiment.

As is seen in Table 4, the majority of features demonstrate a strong or intermediate linear correlation with noise or resolution. Other features demonstrated no correlation with a correlation coefficient, *r* < 0.2. Features that did not correlate with noise or resolution and have a low %COV may be considered highly robust, but also highly insensitive to texture. These features include S.Mean, S.Med, and S.Min.

## Discussion

The aim of this study was to evaluate MRI texture feature repeatability by performing a test-retest study using water and tissue phantoms and evaluate feature robustness by varying the NEX and matrix size. These acquired data were compared with a simulation replicating the impact of the NEX and matrix size on SNR and resolution. The use of phantom data allowed full control of the imaging chain, favouring a better understanding of the relationship between acquisition parameter and feature value.

We found that approximately 80% of MRI texture features are repeatable with a CCC > 0.9 in an immediate test-retest scenario. This result is comparable but less than a recent test-retest CT phantom study, which demonstrated 93.2% of features being repeatable with a CCC > 0.9 [19]. In an MRI phantom study [20], repeatability ranged from 46 to 81% for T1-weighted, T2-weighted, and fluid-attenuated inversion-recovery images, with the highest repeatability found for high-resolution images. Non-phantom, *i.e*, *in vivo*, CT test-retest studies yield worse results, for example, a CT test-retest study of lung tumours after a 15-min interval [21] yielded only 66/219 features (30.1%) as repeatable using the same CCC cutoff (> 0.9). CT test-retest data in a cohort of patients with rectal cancer [22] yielded only 9/542 features (1.7%) with a CCC > 0.85. In a cohort of patients with lung cancer however, 446/542 features were repeatable. Similarly, data from positron emission tomography of oesophageal tumours [23] demonstrated that only half (12/24) of features demonstrated an intraclass correlation coefficient > 0.9 between test and retest with a time interval of 2 days. The ICC and CCC are commonly applied measures of agreement for continuous data. Both measures determine agreement between 2 or more measurements of the same quantity and are useful when assessing test-retest reliability. Values approach 1 when there is near perfect agreement and 0 if no agreement [24]. Like the Pearson correlation coefficient, the ICC assumes a linear relationship between variables; however, the ICC also accounts for the agreement between measurements and is defined as a ratio of subject to total variance using one-way analysis of variance (ANOVA). The CCC assesses both precision and accuracy and evaluates the extent to which pairs of observations fall across the 45° line through the origin [25]. In practice, the values yielded are often similar when using versions of either measure [26].

There is a paucity of MRI texture feature test-retest data, although one study did assess intra-individual repeatability in patients with glioma [27] and found that only 37.0% (386/1043) MRI texture feature were reproducible. In a recent study evaluating repeatability in prostate MRI [28], the authors found that feature repeatability varies greatly and is highly influenced by the pre-processing configuration.

We used a short test-retest interval (less than 5 min) and employed a static phantom. Both of these are likely to improve repeatability compared with patient data, in which motion and more importantly repositioning effects are encountered. For example, PET acquisitions typically last tens of minutes [21] and are therefore highly susceptible to motion, so that image averaging results in motion blurring. Furthermore, we used a fixed ROI and did not segment the data prior to deriving texture features, reducing the effect of motion and positioning on texture feature repeatability.

We recognise that in clinical practice and prospective validation trials, scanner hardware and software variation, changes in acquisition parameters, target lesion motion, segmentation, and ROI placement will degrade feature repeatability. Although it was not the aim of this study to address the impact of scanner and site variability on radiomic data, these data may serve as a benchmark for future radiomic MRI studies investigating these factors. An approach using feature re-alignment and harmonisation, as shown in the recent paper by Orlhac et al. [29] may also help overcome the challenge of multi-centre variability in MRI radiomic data.

In the future, we aim to extend this preliminary study by imaging human volunteers and assessing texture feature repeatability for common tissue types. We expect that texture feature repeatability will be worse in this context compared to the current study and would serve as a limit for repeatability in the clinical setting.

We estimated texture feature robustness by evaluating the linear correlation of texture features with acquisition parameters, and separately the %COV. We found that approximately one-third of features demonstrated low robustness (%COV greater than 30%) and were insensitive to noise or resolution (see Table 3), and three features (skewness, cluster shade, and contrast) were insensitive to both noise and resolution. With regards to correlation with feature value, 5/46 features (10.9%) were poorly correlated (*r* < 0.2) with either noise or resolution (energy, maximum, clustershade, correlation, and low grey-level run emphasis), while three features were poorly correlated (*r* < 0.2) with both noise and resolution (mean, median, and root mean squared).

In a CT phantom experiment, from 43 to 89% of features were found to be reproducible when pitch factor and reconstruction kernel were varied [19]. With regard to MRI texture features, Mayerhoefer et al. [30] looked at sensitivity of texture features with different acquisition parameters in a phantom model and found that NEX, repetition time, echo time, and sampling bandwidth influenced texture features, although this effect was lower at higher spatial resolutions. Becker et al. [31] looked at nonlinear correlations of 19 GLCM- and GLRLM-derived texture features computed from clinical diffusion-weighted sequences of the abdomen with 16 *b* values and found that most texture features were significantly correlated with *b* value. Brynolfsson et al. [32] reported that 19 GLCM-derived texture features from apparent diffusion coefficient maps of glioma and prostate cancer data sets are sensitive to noise, resolution, apparent diffusion coefficient map reconstruction, grey-level quantisation method, and number of grey levels.

The “imresize” function was chosen to reduce spatial resolution in the simulation data. In this study, bicubic interpolation was used, although the function allows for specifying other interpolation methods including nearest-neighbour and bilinear. Bicubic interpolation may retain tissue contrast better; however, it is possible that out-of-range pixel values will be computed due to overshoot as it uses a third-order polynomial [33]. It is likely that feature values would change, should a different function have been utilised. For example, there is existing data that voxel resampling method impacts feature values with linear interpolation resulting in the narrowest feature range, followed by cubic interpolation, whereas nearest neighbour interpolation had the widest range [34].

In this study, there were mixed agreement between the acquired and simulated data, with better agreement for the resolution comparisons than the noise simulation. Discordance in the noise data may be explained by the method used to apply noise in the simulation. This required that additional noise be applied to the base-acquired high SNR image (NEX = 32). Therefore, the noise present in the simulation was not equal to the acquired noise, even though NEX was equal to 32 in both cases.

We recognise a number of limitations of this study. For brevity, some analyses were not performed, for example assessing repeatability of texture features with different contrast weighting or *b* value or the effect of GLCM bin-level. A useful study would be to assess the robustness of texture features to scanner type, vendor, motion, and clinically utilised sequences. To our knowledge this has not been performed. Further, as we have imaged only two phantoms, the variability of underlying textures resulted to be small, and may not be representative of what may be encountered in a clinical setting. As a final limitation of this study, inconclusive results were found between the acquired and simulation data. It has not been possible to entirely account for the source of disagreement between the acquired and simulation data or to gain a full understanding of the relationship between feature class and the relative influence of noise or resolution, for example, why certain features are robust whereas others are not. Of note, the utilisation of simulation data is an original feature of this work and allowed assessment of feature robustness over a range of noise levels and resolutions which may not be easily achievable with data acquired from clinical studies. Finally, we recognise that our results of texture feature robustness cannot be directly translatable to the clinical domain. However, these data should contribute to providing a greater understanding of how texture features behave with MRI acquisition parameters, and in particular multiple acquisitions at different time-points, and should also start to address the broader question of MRI texture feature repeatability, for which currently evidence is lacking.

In conclusion, we have set a limit of repeatability for GLCM- and GLRM-derived MRI texture features, which may serve as a benchmark for further MR studies. Our data demonstrates that robust texture features can be selected for use in clinical radiomic analyses.

## Supplementary Information

**Additional file 1: Supplementary Figure S1.** Resolution response data 1 for tissue phantom (red dots) and water phantom (blue cross) for 46 features. x-axis is resolution level. Feature numbering is described in Table 2. **Supplementary Figure S2.** Noise response data for tissue (red cross) and water phantom (blue dot) for 46 features. NEX Number of excitations. Feature numbering is described in Table 2.

## Data Availability

The datasets used and/or analysed during the current study are available from the corresponding author on reasonable request.

## Abbreviations

%COV: Percentage coefficient of variation
CCC: Concordance correlation coefficient
CT: Computed tomography
GLCM: Grey-level co-occurrence matrix
GLRLM: Grey-level run length matrix
MRI: Magnetic resonance imaging
NEX: Number of excitations
PET: Positron emission tomography
ROI: Region of interest
SD: Standard deviation
SNR: Signal-to-noise ratio
