# Supplementary material for: MRI texture feature repeatability and image acquisition factor robustness, a phantom study and in silico study
Source: Eur Radiol Exp. 2021 Jan 19;5:2. doi: 10.1186/s41747-020-00199-6 (PMC7813908; doi:10.1186/s41747-020-00199-6)
Supplement: Supplementary file 1 — Additional file 1: Supplementary Figure S1. Resolution response data 1 for tissue phantom (red dots) and water phantom (blue cross) for 46 features. x-axis is resolution level. Feature numbering is described in Table 2. Supplementary Figure S2. Noise response data for tissue (red cross) and water phantom (blue dot) for 46 features. NEX Number of excitations. Feature numbering is described in Table 2. [file 41747_2020_199_MOESM1_ESM.docx]

**ELECTRONIC SUPPLEMENTARY MATERIAL**


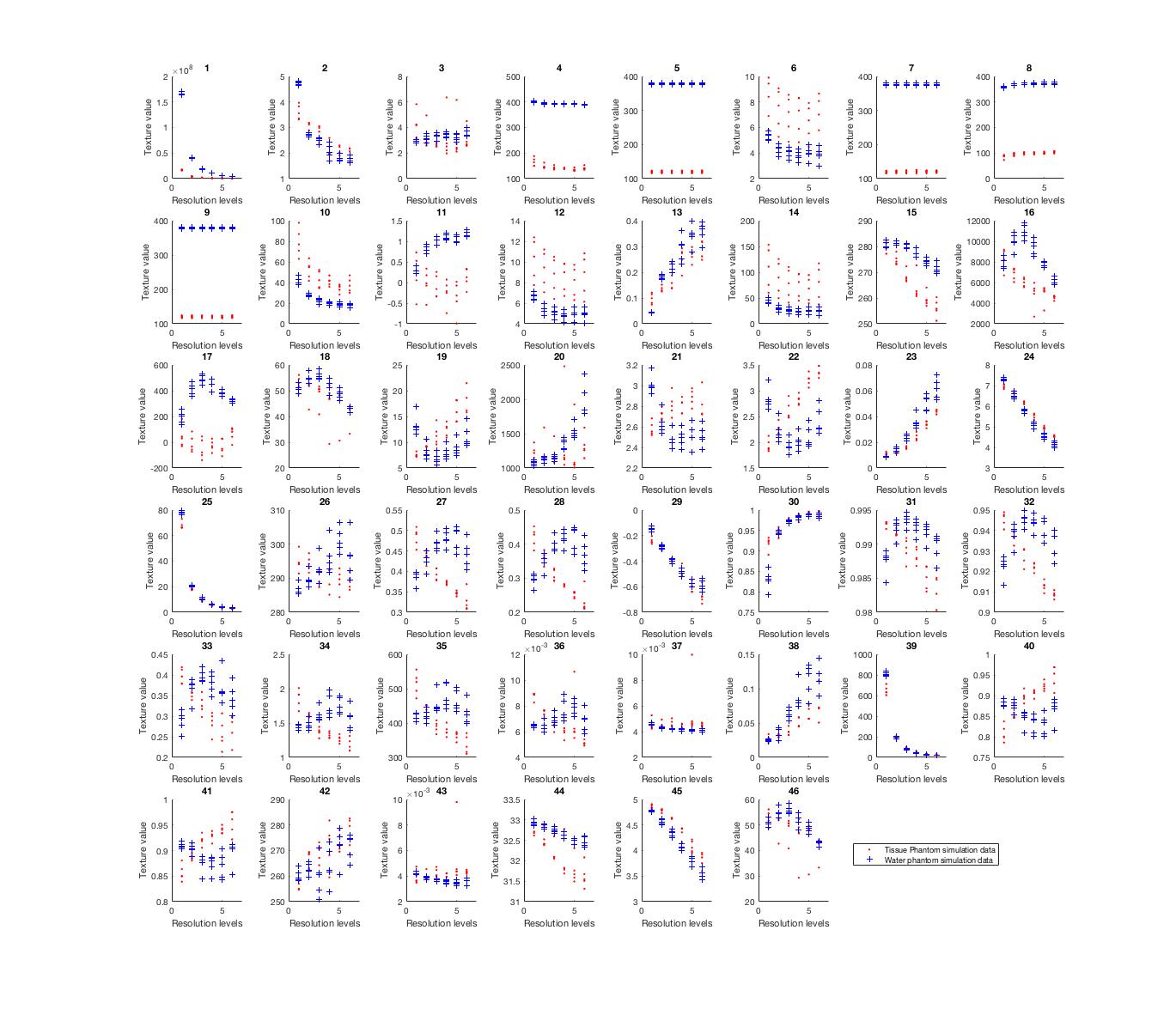


**Supplementary Fig. S1.** Resolution response data 1 for tissue phantom (red dots) and water phantom (blue cross) for 46 features. *x*-axis is resolution level. Feature numbering is described in Table 2.


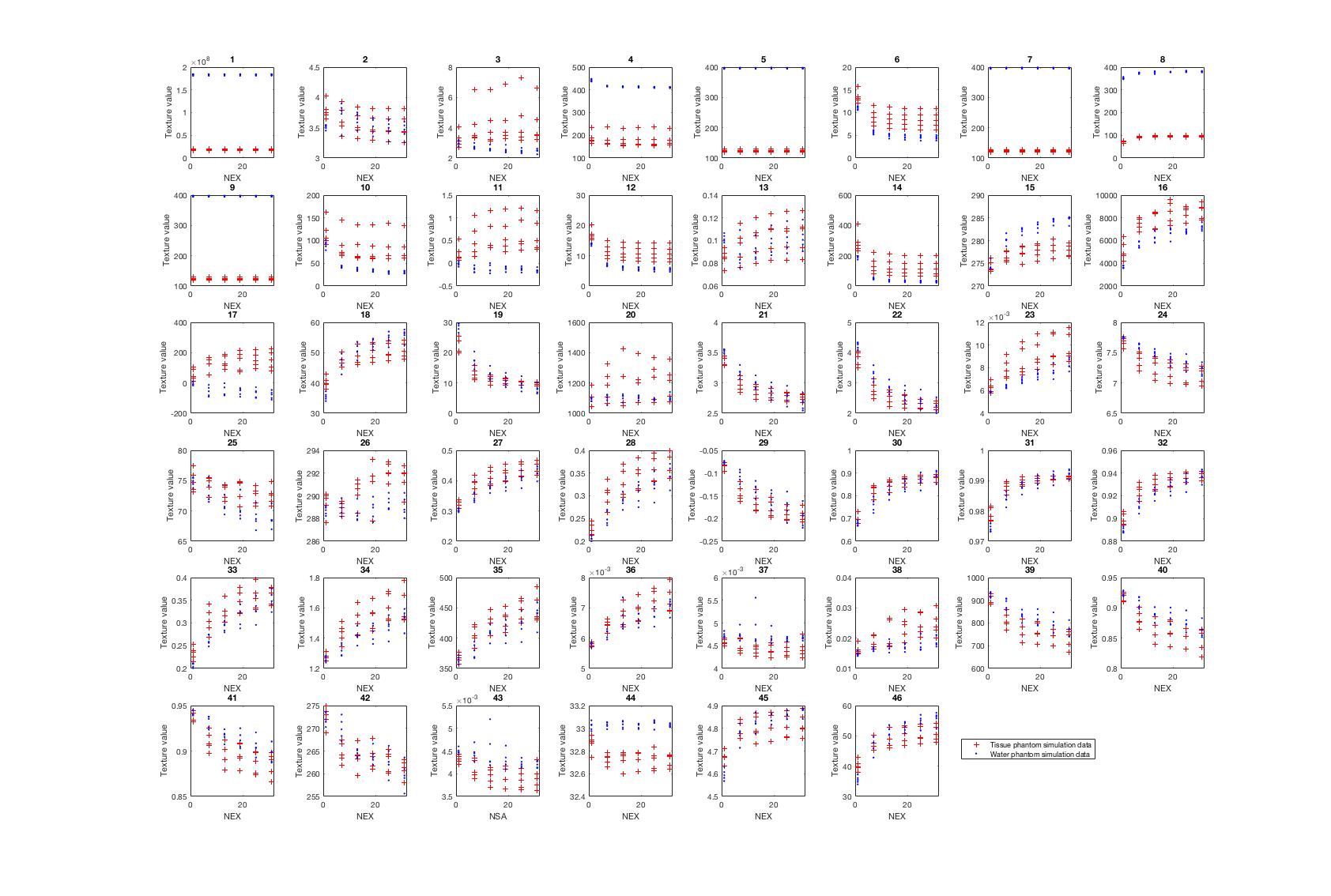


**Supplementary Fig. S2.** Noise response data for tissue (red cross) and water phantom (blue dot) for 46 features. *NEX* Number of excitations. Feature numbering is described in Table 2.
